# Effect of CFTR Modulators on Oxidative Stress and Autophagy in Non-CFTR-Expressing Cells

**DOI:** 10.3390/ijms251910360

**Published:** 2024-09-26

**Authors:** Filippo Scialò, Gustavo Cernera, Lorenza Polise, Giuseppe Castaldo, Felice Amato, Valeria Rachela Villella

**Affiliations:** 1 Dipartimento di Medicina Molecolare e Biotecnologie Mediche, Università di Napoli Federico II, 80131 Napoli, Italy; gustavo.cernera@unina.it (G.C.); lo.polise@studenti.unina.it (L.P.); giuseppe.castaldo@unina.it (G.C.); 2 CEINGE-Biotecnologie Avanzate Franco Salvatore, 80145 Naples, Italy; valeria.villella@gmail.com

**Keywords:** cystic fibrosis, CFTR, Trikafta, ETI, autophagy, Rab5, Rab7

## Abstract

The triple combination therapy for cystic fibrosis (CF), including elexacaftor, tezacaftor and ivacaftor (ETI or Trikafta), has been shown to improve lung function and reduce pulmonary exacerbations, thereby enhancing the quality of life for most CF patients. Recent findings suggest that both the individual components and ETI may have potential off-target effects, highlighting the need to understand how these modulators impact cellular physiology, particularly in cells that do not express CF transmembrane conductance regulator (CFTR). We used HEK293 cells, as a cell model not expressing the CFTR protein, to evaluate the effect of ETI and each of its components on autophagic machinery and on the Rab5/7 components of the Rab pathway. We firstly demonstrate that the single modulators Teza and Iva, and the combinations ET and ETI, increased ROS production in the absence of their target while decreasing it in cells expressing the CFTR ∆F508del. This increase in cellular stress was followed by an increase in the total level of polyubiquitinated proteins as well as the p62 level and LC3II/LC3I ratio. Furthermore, we found that ETI had the opposite effect on Rabs by increasing Rab5 levels while decreasing Rab7. Interestingly, these changes were abolished by the expression of mutated CFTR. Overall, our data suggest that in the absence of their target, both the individual modulators and ETI increased ROS production and halted both autophagic flux and plasma membrane protein recycling.

## 1. Introduction

Cystic fibrosis (CF) is a chronic, progressive, and life-limiting genetic disorder that primarily affects the lungs and digestive system. It is caused by mutations in the cystic fibrosis transmembrane conductance regulator (*CFTR*) gene, which encodes a protein that in the lungs is responsible for regulating the movement of chloride ions out of cells. Although more than 2000 mutations have been identified in the CFTR gene, the most common is the deletion of the amino acid phenylalanine in position 508 (CFTR-∆F508del) [1,2,3]. This defective protein leads to the production of thick, sticky mucus that can clog airways, obstruct the pancreas, and affect other organs. The hallmark symptoms include persistent coughing, frequent lung infections, wheezing, shortness of breath, difficulty in absorbing nutrients, greasy stools, and severe constipation [4,5,6]. Over time, the accumulation of mucus and recurrent infections can cause significant lung damage, leading to respiratory failure, the most common cause of death in CF patients [7].

CFTR modulators, a groundbreaking class of drugs, target the underlying cause of CF by correcting the folding and enhancing the function of the defective CFTR gene. Specifically, the new triple combination, named ETI (or Trikafta), which was initially designed to target CFTR-∆F508, has now been approved for another 178 CFTR mutations [8,9]. ETI is composed of two correctors: (i) elexacaftor (Elexa) and (ii) tezacaftor (Teza), which lead CFTR to plasma membranes (PMs), improving its functionality, and iii) ivacaftor (Iva) a potentiator that increases the flow of chloride ions [10,11]. The efficacy of ETI in improving lung function, reducing pulmonary exacerbations, and enhancing the quality of life for many patients has been demonstrated in clinical trials and previous publications [12,13,14]. Unfortunately, like all drugs, despite the undeniable positive effects, common adverse events have been reported, such as headaches, upper respiratory tract infections, abdominal pain, diarrhea, rashes, increased liver enzymes, metabolic alterations, impact on Beta cell functions, nausea, dizziness, fatigue, and sinus congestion [14,15,16,17,18,19,20]. Furthermore, numerous studies are now indicating that both the individual components and their combinations may have potential off-target effects. For instance, Teza has been shown to inhibit sphingolipid delta-4 desaturase in a concentration-dependent manner, causing an accumulation of dihydroceramides [21,22]. Iva and its metabolites have a significant affinity for serotonin and β3-adrenergic and δ-opioid receptors [23], while ETI has antimicrobial activities against Staphylococcus aureus [24,25]. Altogether, these data suggest that CFTR modulators can have profound repercussions on cellular physiology. Most importantly, the potential off-target effects could be more significant in a cellular environment where the normal target—a mutated CFTR gene—is missing. Studies exploring these effects are scarce, leaving a knowledge gap regarding the possible interactions among the modulators and other cellular machinery. Therefore, to understand if ETI could modulate cellular physiology independently of CFTR, we sought to study both the effect of each component alone and their double and triple combinations on human embryonic kidney (HEK) cells that normally do not express CFTR (referred below as HEK-CFTR null cells) [26]. First, we evaluated whether the treatments with the single modulators, ET, and ETI, had any harmful effects on the cells. Next, we focused on examining how these drugs affected two crucial and interconnected processes: the autophagy machinery and the Rab5/7 components of the Rab pathways. These processes play essential roles in housekeeping functions, including the removal of misfolded or aggregated proteins, recycling of plasma membrane (PM) proteins, clearing damaged organelles such as mitochondria, the endoplasmic reticulum, and peroxisomes, and eliminating intracellular pathogens [27]. Previous studies have already demonstrated the role and involvement of autophagy and the Rabs pathway in the regulation of CFTR fate in CF cell lines, showing the effects of ETI treatments on these pathways [28,29,30,31,32,33]. However, to our knowledge, no study has yet examined how ETI or its components might influence these critical pathways in cells that do not express CFTR. Understanding this is crucial, as it could provide insights into whether and how these modulators might modulate these pathways, potentially affecting cellular physiology and organ function.

## 2. Results and Discussion

### 2.1. CFTR Modulators Increase Reactive Oxygen Species and Autophagic Markers in CFTR-Null HEK Cells

**Figure 1:**
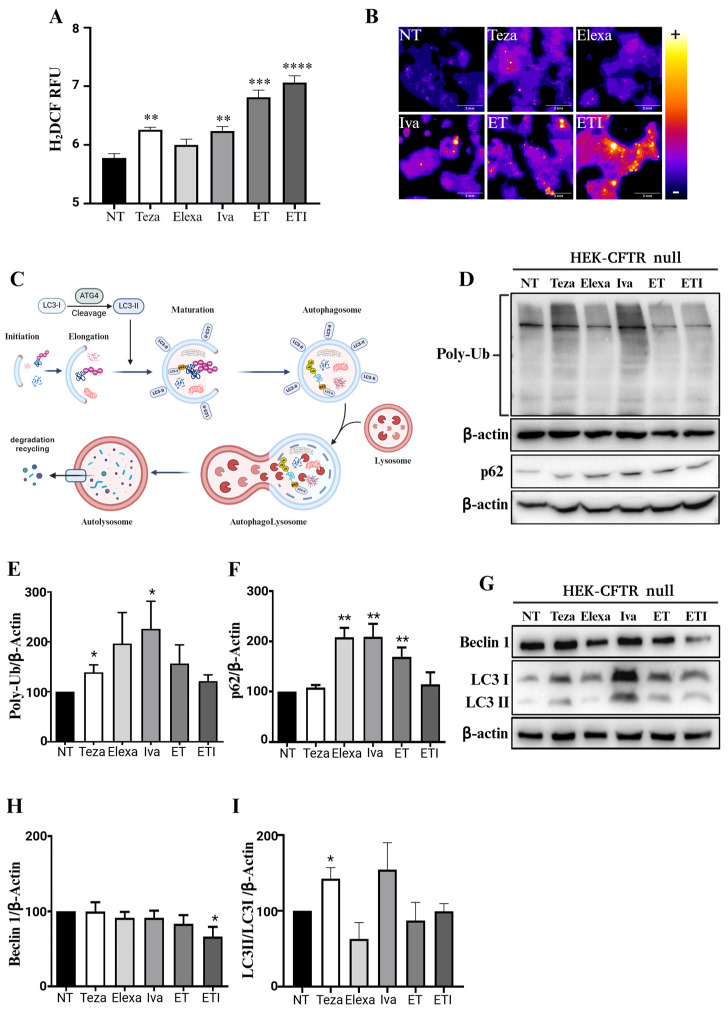
Effect of CFTR modulators on ROS production and autophagy in HEK-CFTR null cells. (**A**) The cells were stimulated with Teza (10 µM), Elexa (3 µM), and Iva (5 µM) alone or in combinations (ET and ETI) for 24 h and compared with control/unstimulated cells (NT). The H_2_DCF staining has been evaluated. The data are expressed as means ± SEM (*n* = 3; (**A**) NT vs. Teza ** *p*: 0.0013; NT vs. Iva ** *p*: 0.0054; NT vs. ET *** *p*: 0.0002; NT vs. ETI **** *p*: 0.0001). (**B**) Microphotographs are representative of at least three independent experiments. (**C**) A representative schema presents the main steps of the autophagic process. (**D,I**) The expression of PolyUb, p62, Beclin-1 and LC3-I/II has been evaluated by western blot. β-actin levels are presented as an internal control for the experiment. In (**D**,**G**), representative images of 3 independent experiments are shown. In (**E**,**F**,**H**,**I**), the quantification of the western blots is shown; the data are expressed as means ± SEM (*n* = 3; (**E**) NT vs. Teza * *p*: 0.0283; NT vs. Iva * *p*: 0.0424; (**F**) NT vs. Elexa ** *p*: 0.001; NT vs. Iva ** *p*: 0.005; NT vs. ET ** *p*: 0.009; (**H**) NT vs. ETI * *p*: 0.021; (**I**) NT vs. Teza * *p*: 0.017).

We hypothesized that the modulation of any cellular process by Elexa, Teza, Iva, ET, or ETI would be more pronounced in cells that do not naturally express CFTR. This is physiologically relevant because not all cells in the body express CFTR, and the influence of these drugs in a cellular environment lacking their mutated target has not been deeply investigated yet. Therefore, to test if CFTR modulators or their combinations could be linked to any unintended cellular effect, we sought to examine if they had an impact on intracellular redox status. An increase in reactive oxygen species (ROS) production is generally linked to a state of oxidative stress, which has significant implications for cell health and function. Oxidative stress occurs when there is an imbalance between the production of ROS and the cell’s ability to detoxify these reactive intermediates or repair the resulting damage. Elevated ROS levels can lead to oxidative damage to cellular components, including lipids, proteins, and DNA [34]. We measured ROS production in HEK-CFTR null cells treated with the single CFTR modulators or their combinations by staining them with H_2_DCF, an ROS indicator. Unexpectedly, HEK-CFTR null cells treated with Teza, Iva, ET, and ETI had more ROS compared to the untreated control cells or those treated with Elexa (Figure 1A,B). This increase in oxidative stress suggested a potential negative impact on cellular physiology, possibly inducing lipid peroxidation, DNA and protein damage, mitochondrial dysfunction, and inflammation [34]. Moreover, this increased production of ROS can have a profound impact on autophagy, a crucial cellular process involved in maintaining homeostasis and responding to stress by recycling damaged organelles, misfolded proteins, and other intracellular debris (Figure 1C). Therefore, to assess if CFTR modulators would also impact on the autophagic process, we assessed the level of autophagy markers in HEK-CFTR null cells treated with CFTR modulators. Teza and Iva caused a significant increase in the total levels of polyubiquitinated (PolyUb) proteins, while an increasing trend was seen with Elexa (Figure 1E,F). p62/SQSTM1 (referred to below as p62) which acts as an autophagy receptor [35], recognizing and binding to ubiquitinated proteins and organelles that need to be degraded, was increased with Elexa, Iva and ET (Figure 1D,F). Interestingly, the increases in PolyUb and p62 were completely abolished when the drugs were used in combination (Figure 1D–F). Furthermore, we observed a small but significant reduction in Beclin-1 levels in cells treated with ETI (Figure 1G,H). Additionally, like the increases seen in PolyUb and p62, the ratio of LC3II/LC3I used to assess the autophagic flow was also increased in cells treated with Teza and Iva (Figure 1G,I). To confirm the effect of CFTR modulators on the autophagic flux we treated the cells with Bafilomycin A1, an inhibitor of vacuolar H+-ATPase, commonly used to block autophagy in its late phase [36]. CFTR modulators with or without bafilomycin A1 were used to treat HEK-CFTR null cells and, as shown in Supplementary Figure S1A–C, besides ETI, all modulators were able to increase the LC3II/LC3I ratio.

### 2.2. ET and ETI Modulate Rab5 and Rab7 in HEK-CFTR Null Cells

**Figure 2:**
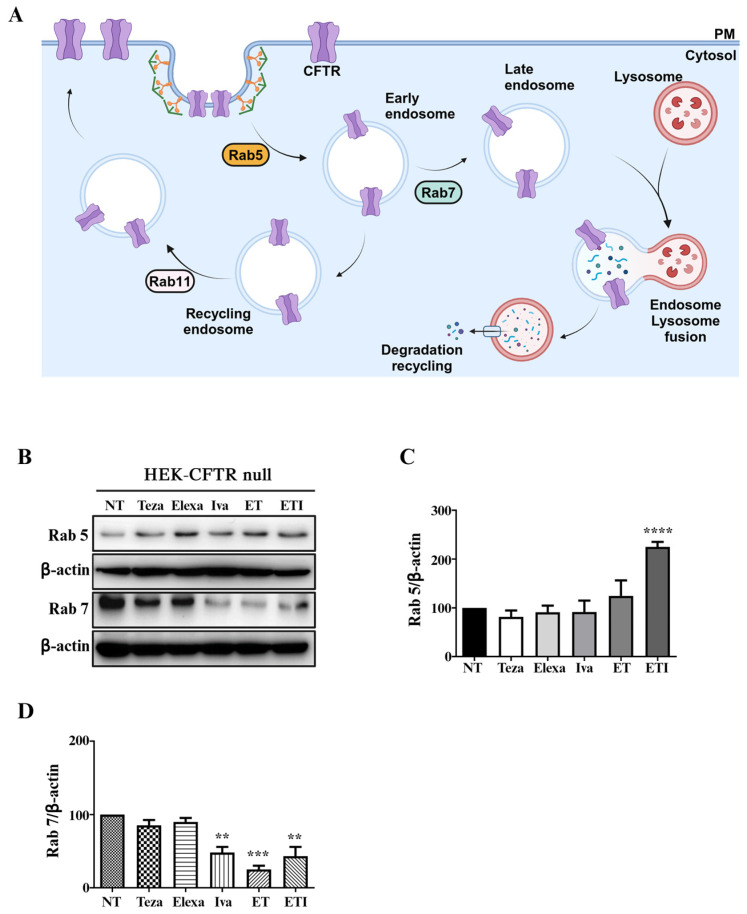
Effect of CFTR modulators on Rab5 and Rab7 expression in HEK-CFTR null cells. (**A**) The figure schematically illustrates the role of the Rab family in vesicle transport, recycling and protein degradation. (**B**–**D**) The cells were stimulated with Teza (10 µM), Elexa (3 µM), and Iva (5 µM), alone or in combinations (ET and ETI), for 24 h and compared with control/unstimulated cells (NT). The expression of Rab5 and Rab7 has been evaluated by western blot. β-actin levels are presented as an internal control for the experiment. In (**B**), the images are representative of at least three experiments yielding similar results. In (**C**,**D**), the relative quantifications are shown; the data are expressed as means ± SEM (*n* = 3; (**C**) NT vs. ETI **** *p*: <0.0001; (**D**) NT vs. Iva ** *p*: 0.026; NT vs. ET *** *p*: 0.0003; and NT vs. ETI ** *p*: 0.0094).

Rab GTPases belong to the Ras superfamily, with more than 60 members that can reversibly associate with intracellular membranes regulating a wide range of functions linked to vesicle transport, recycling, and protein degradation [27]. Regulation of CFTR at the PM involves two key processes: modulating the chloride channel activity and controlling the amount of channel present at the PM at any given moment. CFTR recycling is achieved through endocytosis via clathrin-coated vesicles, which either recycle the channel back to the PM or direct it to lysosomal degradation. These trafficking events are coordinated by several protein partners, including Rab GTPases [27] (Figure 2A). Specifically, Rab5 directs CFTR transport from the PM to endocytic vesicles and fusion with early endosomes, while Rab7 mediates early-to-late endosome and late endosome-to-lysosome transport (Figure 2A) [37]. By examining the effect of the modulators on HEK-CFTR null cells, we found that ETI significantly increased the level of Rab5, whereas the single modulators showed no effect (Figure 2B,C). Interestingly, when Rab7 was assessed, the opposite trend was observed. The treatments with Iva, ET, and ETI significantly decreased Rab7 levels, whereas Teza and Elexa showed no significant changes (Figure 2B,D). Similar to the findings related to autophagy, these results imply that Iva, ET, and ETI may halt PM protein turnover. 

### 2.3. CFTR Modulators Do Not Affect Autophagy in CFTR-∆F508del HEK Cells

**Figure 3:**
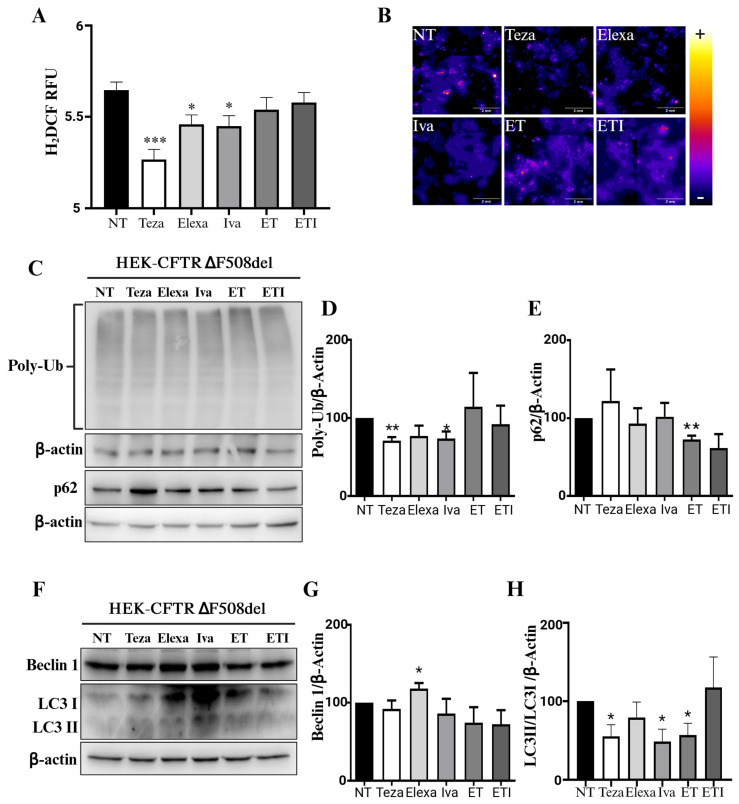
Effect of CFTR modulators on ROS production and autophagy in Hek-CFTR-∆F508del cells. ROS production**,** PolyUb, and Beclin-1 levels, as well as LC3 maturation and p62 degradation, were evaluated in HEK-CFTR-∆F508del cells in the same experimental conditions shown in Figure 1: treatment with Teza (10 µM), Elexa (3 µM), Iva (5 µM) alone or in combinations (ET and ETI) for 24 h and compared with control/unstimulated cells (NT). β-actin levels are presented as the internal control for the experiment. (**A**) The H2DCF staining has been evaluated. The data are expressed as means ± SEM (*n* = 3; (**C**) NT vs. Teza *** *p*: 0.0003; NT vs. Elexa * *p*: 0.0223; NT vs. Iva * *p*: 0.0228). Microphotographs in (**B**) and data in (**C**,**F**) are representative of at least three independent experiments. In (**D**,**E**,**G**,**H**), the relative quantifications are shown; the data are expressed as means ± SEM (*n* = 3; (**D**) NT vs. Teza ** *p*: 0.0032; NT vs. Iva * *p*: 0.0447; (**E**) NT vs. ET ** *p*: 0.0056; (**G**) NT vs. Elexa * *p*: 0.0314; (**H**) NT vs. Teza * *p*: 0.0109; NT vs. Iva * *p*: 0.011; and NT vs. ET * *p*: 0.0192).

To link the use of modulators with the observed increase in cellular oxidative stress and autophagic markers we used HEK-CFTR-∆F508del cells previously produced in our laboratory [26]. We sought to discover whether the expression of this mutated CFTR channel would reduce the likelihood of nonspecific binding of the modulators to other targets, thereby diminishing their off-target effect. If these compounds effectively interact with the defective CFTR caused by the ∆F508del mutation, their modulatory effects on oxidative stress and autophagy markers should be diminished or negated. Using the HEK-CFTR-∆F508del cells, we confirmed that the modulators behaved in opposite manners. Teza, Elexa, and Iva decreased ROS production, while ET and ETI caused no change compared to the untreated control cells (Figure 3A,B), further confirming the differing actions of these modulators when their target is absent. The autophagic markers were also differently regulated. Treatment with Teza and Iva, which previously caused the largest increase in total PolyUb levels in the absence of CFTR, significantly decreased it in the presence of CFTR-∆F508del (Figure 3C,D). Conversely, the combinations of modulators had no effect. A similar pattern was observed for p62 levels: Elexa, Iva, and ET increased p62 levels in HEK-CFTR null cells, whereas a slight decrease was noted in HEK-CFTR-∆F508del cells treated with ET (Figure 3C,E). Beclin-1 was the least affected, increasing only after Elexa treatment (Figure 3F,G). Contrary to their effects on HEK-CFTR null cells, Teza, Iva, and Elexa slightly decreased the LC3II/LC3I ratio in HEK-CFTR-∆F508del cells (Figure 3F,H). Again, CFTR-∆F508del cells treated with bafilomycin A1 showed that Teza, Elexa, Iva, and ET increased LC3II/LC3I ratios compared with the non-treated control cells, while ETI did not (Supplementary Figure S1D–F).

### 2.4. Rab5 and Rab7 Are Differently Regulated in HEK-CFTR-∆F508del Cells Treated with CFTR Modulators

**Figure 4:**
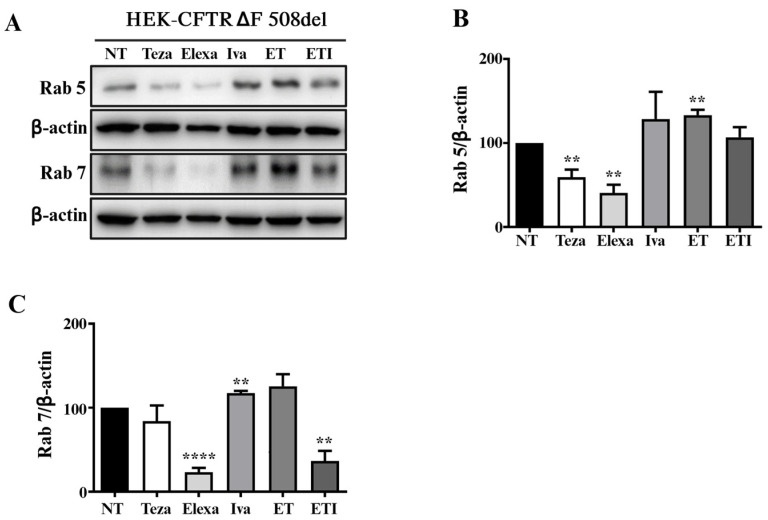
Effect of CFTR modulators on Rab5 and Rab7 expression in HEK-CFTR-∆F508del cells. (**A**–**C**) The cells were stimulated with Teza (10 µM), Elexa (3 µM), and Iva (5 µM), alone or in combinations (ET and ETI), for 24 h and compared with control/unstimulated cells (NT). The expression of Rab5 and Rab7 has been evaluated by western blot. β-actin levels are shown as an internal control for the experiment. In (**A**), the images are representative of at least three independent experiments. In (**B**,**C**) the relative quantifications are shown; the data are expressed as means ± SEM (*n* = 3; (**B**) NT vs. Teza ** *p*: 0.008; NT vs. Elexa ** *p*: 0.0037; NT vs. ET ** *p*: 0.0056; (**C**): NT vs. Elexa **** *p*: 0.0003; NT vs. Iva ** *p*: 0.0034; and NT vs. ETI ** *p*: 0.006).

As previously observed with autophagic markers, the regulation of the Rab5 and Rab7 pathways also changed based on the presence of the mutated CFTR. Specifically, Teza and Elexa, which did not affect HEK-CFTR null cells, decreased Rab5 and Rab7 expression levels in HEK-CFTR-∆F508del cells (Figure 4A–C). Additionally, Iva and ET increased the levels of both markers, but decreased Rab7 levels when CFTR was not expressed. ETI treatment further demonstrated distinct effects; it did not alter Rab5 levels but decreased Rab7 levels in the presence of ∆F508del expression (Figure 4A–C).

Therefore, by using HEK-CFTR null cells, which do not express CFTR, we have demonstrated that Teza, Iva, ET, and ETI, but not Elexa, play a harmful role by inducing an increase in cellular ROS levels (Figure 1A,B). Interestingly, the same compounds induced a decrease when the mutated form of CFTR-∆F508del was expressed (Figure 3A,B). It is well known that cells can produce ROS through various enzymes located in different cellular compartments. For example, there are at least eleven sites of ROS production within the mitochondria alone [38], and numerous others in the cytoplasmic compartment [39]. The presence of exogenous molecules such as Teza or Iva could potentially interfere with some of these ROS-producing enzymes or bind to other proteins that do not normally induce ROS but may trigger their generation under these conditions. On the contrary, Elexa probably does not produce an increase in ROS because the protein/enzyme it potentially binds to cannot cause this increment. Identifying the mechanism by which these modulators induce oxidative stress would be pivotal to a better comprehension of how Trikafta impacts cellular physiology.

The increase in oxidative stress seen in HEK-CFTR null cells was accompanied by an increase in autophagic markers. We demonstrated that Teza and Iva increased PolyUb levels (Figure 1D,E), while Elexa, Iva, and ET raised the levels of p62 (Figure 1D,F). Interestingly, these effects were completely abolished when the drugs were used in combination. These increases seen in PolyUb and p62 levels were mirrored by the increase in the LC3II/LC3I ratio, which was maintained when cells were treated with bafilomycin A1 (Figure 1G,I and Supplementary Figure S1A–C). Although an increase in the LC3II/LC3I ratio would point towards an increase in the autophagic flux, this was not supported by a decrease in p62, which, on the contrary, was increased when cells were treated with Elexa, Iva, and ET, suggesting instead a block in the autophagic process. Furthermore, we found that ETI significantly increased the level of Rab5 (Figure 2B,C), while Rab7 was decreased by Iva, ET, and ETI (Figure 2B,D). These data suggest that, as in the case of autophagy, the PM protein recycling operated by Rab5/7 might also be halted, especially by ETI. A hypothesis that might explain the halting of autophagy and Rab pathways may be suggested by the physical binding of these modulators to other proteins. It has been well described that the modulators’ actions are dictated by their physical binding within specific CFTR pockets [40,41,42,43]. Moreover, as previously discussed, several studies have shown that these modulators exhibit affinity for other targets, influencing their functions [14,15,16,17,18,19,20,21,22,23,24,25]. This implies that these molecules could physically bind to other proteins/enzymes that might have a similar pocket in their 3D structures. Therefore, if these modulators can bind to other proteins even in the presence of their target—mutated CFTR—we anticipate that off-target effects would be exacerbated in CFTR null cells, where the modulators, now unable to bind to CFTR, would be free to interact with other targets.

Therefore, to directly link the use of modulators to the observed off-target effects and confirm their role in these processes, we expressed CFTR-∆F508del. The presence of this mutated target completely abolished the increase in ROS production seen in the HEK-CFTR null cells (Figure 3A). The same effect was shown for the autophagic and Rab markers. Teza and Iva, which increased PolyUb and p62 levels in HEK-CFTR null cells, had an opposite effect in HEK-CFTR-∆F508del cells by decreasing their level (Figure 3C–E). The same effect was observed for the LC3II/LC3I ratio, which increased in the absence of CFTR with Teza, Iva, and Elexa, but decreased in HEK-CFTR-∆F508del cells. As shown in Supplementary Figure S1D–F, besides ETI, bafilomycin A1 treatment increased the LC3II/LC3I ratio compared to the respective non-treated control cells. Again, here the total level of PolyUb protein did not change and the p62 level showed only a slight decrease after ET treatment, suggesting that the autophagic flux was similar to the untreated HEK-CFTR-∆F508del cells.

As observed with autophagic markers, we found that the regulation of Rab5/7 was dependent on the presence of the mutated form of CFTR. Specifically, Teza and Elexa, which did not affect HEK-CFTR null cells (Figure 2), decreased the levels of Rab5/7 in HEK-∆F508del cells (Figure 4). Similarly, Iva and TE increased the levels of both markers, while decreasing Rab7 when CFTR was absent. ETI treatment also showed differences: in the presence of ∆F508del expression, it did not change Rab5 levels but decreased Rab7 expression. The data presented here add to an existing body of evidence demonstrating the impact that the off-target effects of these modulators have on cellular physiology. It is important to note that patients often experience numerous side effects, as highlighted in the Trikafta leaflet [44], which can also involve other organs than the lungs [45,46]. Our results suggest that these effects are clinically relevant because, over time, increased oxidative stress and the blocking of autophagy/Rab pathways can lead to cellular damage, loss of normal cellular function, and potential organ dysfunction. This study presents the following limitations: (i) it is focused on specific pathways and may not address other relevant mechanisms involved, (ii) it was conducted over a limited timeframe, which may not capture long-term effects, (iii) the results are specific to the cells used in this study and attempts should be made to replicate them in other cell types.

## 3. Materials and Methods

### 3.1. Cell Culture and Generation of HEK293 Cells Expressing the F508del-CFTR

Cell cultures of HEK-CFTR null cells and HEK-CFTR-∆F508del cells were maintained as previously described by Esposito et al. [26]. Briefly, HEK-CFTR null cells were cultured in DMEM (Dulbecco’s Modified Eagle Medium) supplemented with 10% FBS (fetal bovine serum), 100 U mL^−1^ of penicillin-streptomycin (Pen-Strep), 1 mM of sodium pyruvate and 2 mM of L-glutamine (all from Euroclone, Milan, Italy) in a 37 °C, 5% CO_2_ humidified incubator. HEK-CFTR null cells were transduced with lentiviral particles produced from HEK293T cells previously transfected with recombinant plasmids of YFP-CFTR-F508del. The mix of 2.5 μg of pRSV-REV, 3.5 μg of pMD2.G, 6.5 μg of pMDLg/pRRE and 10 μg of YFP-CFTR-F508del in a final volume of 450 μL with 0.1XTE and 50 μL of 2.5 M CaCl_2_, were used to transfect 5 × 10^6^ cells in a 10 cm dish. The medium was replaced after 6 h and lentiviral particles were collected 48 h after transfection.

### 3.2. Cell Treatments

HEK-CFTR null and HEK-CFTR-F508del cells were seeded in a six-well plate at 1 × 10^6^ cell\plate or in a 96-well plate at 1 × 10^4^ cell\plate in complete medium and after 24 h the cells were treated with tezacaftor (10 μM), elexacaftor (3 μM), ivacaftor (5 μM) or a combination of ET or ETI for 24 h. At the end of treatments, the cells were harvested and lysed for western blot analysis or ROS detection.

### 3.3. Western Blot

HEK-CFTR null cells and HEK-CFTR-F508del cells were treated as described in the previous paragraph; at the end of treatments, the cells were harvested and lysed for western blot analysis. Forty micrograms of total extract were used for SDS-polyacrylamide gel electrophoresis. The PVDF membranes were blocked with 5% non-fat dry milk and incubated O\N with primary antibodies: Beclin-1 (1:1500, ab207612, Abcam, Cambridge, UK); Rab-5 (1:1000, ab18211, Abcam, Cambridge, CB2 0AX, UK); Rab-7 (1:1000, ab77993, Abcam, Cambridge, CB2 0AX, UK), LC3B (1:1500, ab51520, Abcam,Cambridge, CB2 0AX, UK); Ubiquitin P4D1 (1:1000, #3936, Cell signaling, Danvers, Massachusetts, (USA)); p62/SQSTM1 (1:1500, p0067, Merck, Milan, Italy); and β-actin monoclonal (1:25,000, A3854, Merck, Milan, Italy). Secondary antibodies with horseradish peroxidase (HRP)-conjugated (1:8000 in 5% non-fat dry milk, Elabscience, Houston, Texas, 77079, USA) were incubated for 1 h at RT and signals were finally detected with a chemiluminescent detection system by using the Chemidoc MP Imaging System Biorad (Milan, Italy).

### 3.4. Reactive Oxygen Species Detection

After treatments, HEK-CFTR null cells and HEK-CFTR-∆F508del cells were washed with PBS and assayed with a Fluorometric Intracellular ROS kit (MAK142-1KT, Merck) following the manufacturer’s instructions. The images were captured with Cell Discoverer 7, (Zeiss, Wetzlar, Germany) and analyzed with Image J software (free download software, Rasband, W.S., ImageJ, U.S. National Institutes of Health, Bethesda, MD, USA, https://imagej.net/ij/, 1997-2018).

### 3.5. Statistical Analysis

To evaluate the differences in mean values between each experimental group and the control group, an independent unpaired *t*-test was performed. The data are representative of at least three independent experiments and are expressed as means ± SEM. *p* < 0.05: * *p* < 0.01: ** *p* < 0.001: *** *p* < 0.0001: ****.

## 4. Conclusions

Emerging evidence have demonstrated off-target effects of ETI and its components. For instance, Teza can lead to dihydroceramide accumulation, which is associated with cardiovascular diseases and obesity-related conditions like type 2 diabetes and atherosclerosis [47,48,49,50]. Iva can bind to receptors involved in neurological functions, suggesting it may affect brain activity in CF patients [51,52]. Here, we demonstrate that individual modulators and their combinations increased ROS production and halted autophagic flux and the Rab5/7 pathway in HEK cells, which do not normally express CFTR. This finding is corroborated by the expression of the mutated form of CFTR-∆F508del, which can diminish the modulators’ effects. Our data indicate that the off-target effects of CFTR modulators can negatively impact cellular physiology, especially in those cells where their mutated target is absent.

## Data Availability

The original contributions presented in the study are included in the article/Supplementary Materials, further inquiries can be directed to the corresponding author.

## Supplementary Materials

The following supporting information can be downloaded at https://www.mdpi.com/article/10.3390/ijms251910360/s1.
