# Supplementary material for: Effect of CFTR Modulators on Oxidative Stress and Autophagy in Non-CFTR-Expressing Cells
Source: Int J Mol Sci. 2024 Sep 26;25(19):10360. doi: 10.3390/ijms251910360 (PMC11476568; doi:10.3390/ijms251910360)
Supplement: Supplementary file 1 [file ijms-25-10360-s001.zip › ijms-3165134-supplementary.pdf]

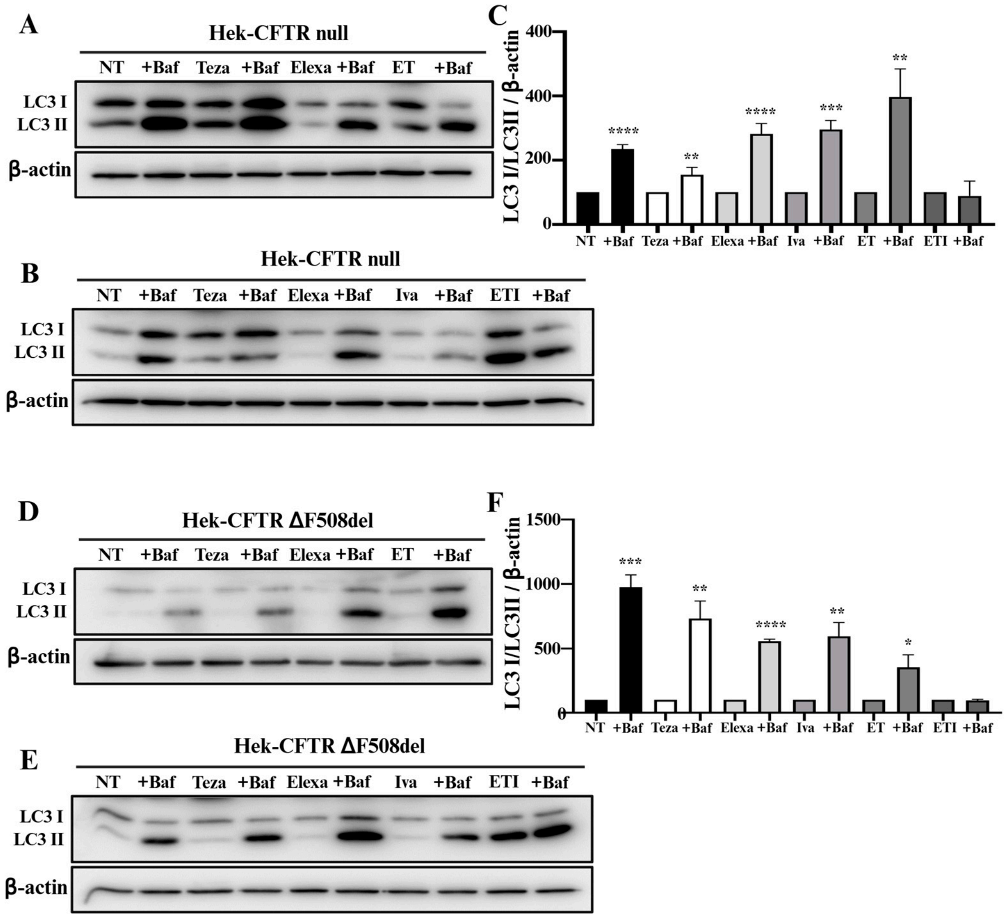


**Supplementary Figure 1**

**Supplementary Figure S1: Effect of CFTR modulators on autophagy in Hek-CFTR null and -ΔF508del cells.** LC3I/II expression was evaluated in CFTR-null and -ΔF508del Hek cells. β-actin levels were assessed as internal control of the experiment. The cells were treated with Bafilomycin A1 (Baf, 1 μM) for 4h. After, the cells were treated with Teza (10 μM), Elexa (3 μM), Iva (5 μM) alone or in combinations (ET, ETI) for 24 hours and compared with control/unstimulated cells (NT). Data in (A, B) are representative of at least three independent experiments. In (C), the relative quantification is shown; the data are expressed as means ± SEM ($n = 3$; C: NT vs. +Baf ****$p < 0.0001$; Teza vs. +Baf **p: 0.0048; Elexa vs. +Baf ****$p < 0.0001$; Iva vs. +Baf ***p: 0.0003; ET vs. +Baf **p: 0.0043). Data in (D, E) are representative of at least three independent experiments. In (F), the relative quantification is shown; the data are expressed as means ± SEM ($n = 3$; C: NT vs. +Baf ***$p < 0.0001$; Teza vs. +Baf **p: 0.0026; Elexa vs. +Baf ****$p < 0.0001$; Iva vs. +Baf **p: 0.0088; ET vs. +Baf *p: 0.0472).
